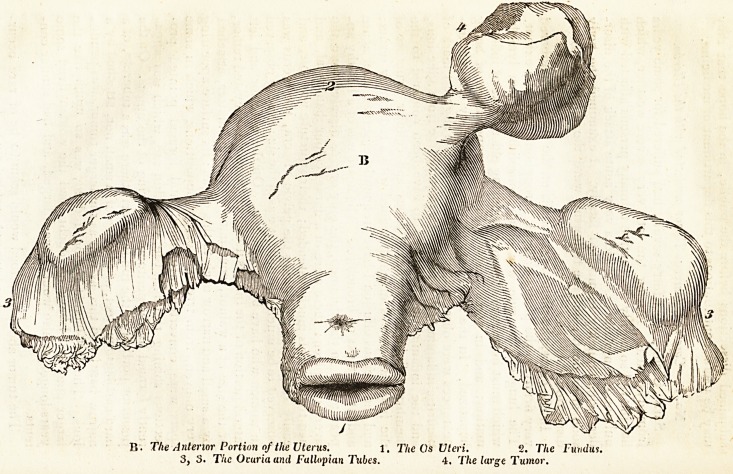# Contributions to Morbid Anatomy

**Published:** 1823-05

**Authors:** R. Wade

**Affiliations:** Member of the Royal College of Surgeons, and Apothecary to the Westminster Dispensary.


ART. VI.—*Contributions to Morbid Anatomy.* By R. WADE, Member of the Royal College of Surgeons, and Apothecary to the Westminster Dispensary.

PATHOLOGY offers a wide field for investigation, embracing, as it does, the nature, causes, and symptoms of disease; and, although daily enriched by new discoveries, it still offers a fruitful soil to the cultivator. From the nature of the science, its improvement must be slow; for here the splendid theories of genius, however brilliant, instead of aiding our progress, will rather, by dazzling the imagination, turn us out of the direct road, which is only to be attained by persevering industry and sound reasoning upon facts. Morbid anatomy is the basis of pathology, and offers these facts to our view. The importance of post-mortem examinations is universally felt and acknowledged; yet such, unhappily, is the prejudice of mankind, that we frequently are prevented, when we most wish it, from ascertaining correctly the nature of disease. It often happens, also, that those individuals who have most opportunities for such examinations are so much occupied by indispensable avocations, that their knowledge (like the pearl at the bottom of the ocean, which throws its lustre only on a few surrounding objects,) is frequently either confined to their own minds, or communicated but to few. From the extensive opportunities for post-mortem examinations at a public dispensary, some circumstances are occasionally developed, that are new even to the most experienced practitioner, and therefore will amply reward him for his labour; but, to the student, any deviation from natural and healthy structure must be of the utmost importance, and every opportunity ought to be embraced and sought after, with a view to his mind being well stored with facts, upon which his after celebrity will depend. Under the influence of this latter consideration, I am induced to lay the subjoined dissections before the public, in the hope that they may be useful contributions to this department of medicine.

No. I.—*Case of Calcareous Tumor connected with the Uterus.*

Sarah Glenn, aged forty-seven, had suffered for some years from dyspnœa. During the late very severe weather, which has proved so highly destructive to many persons affected with pulmonic complaints, she had a very acute attack of the disorder, which quickly terminated fatally. I examined the body soon after death, and the following were the appearances on dissection.

In the chest, the pleura pulmonalis on the right side adhered very firmly to the pleura costalis, throughout its whole extent. The lung on this side was much consolidated from lymph, which must have been thrown out during old attacks of inflammation; and in some parts it had the appearance of being hepatized. In the left cavity of the thorax, very different appearances were observed, there being not the slightest mark of pleuritic inflammation; the lung was quite free from adhesions, and the inflammation appeared to have been confined to the membrane lining the bronchial tubes and cellular structure of the lung, which was much distended with a red frothy serum: and such serous effusion I have frequently observed after acute and fatal attacks of bronchitis. The heart was of a healthy appearance. In the abdomen, the only organs diseased were the liver and spleen: these were of a darker colour than usual, and had their vessels much distended; their structure was soft, and easily torn. On examining the pelvic viscera, the uterus was observed to be much diseased: it was of the size usually observed in women who have borne children. On the anterior and left lateral portion of the fundus uteri, was found an encysted tumor, of the size of a walnut, containing calcareous matter, attached by a small and narrow neck to this organ; its surface appearing very uneven, from irregular earthy depositions, which in some spots were converted into bone, and formed pointed projections shining through the very thin sac, which was apparently formed by the peritoneal covering of the uterus. On the posterior portion of the fundus uteri, there was a tubercle, of a hard cartilaginous feel, of the size of a small nut, attached by a narrow base; and this, as well as the larger tumor, was pendulous. The last-named tumor, indeed, was so much like the larger one, that I have no doubt, had the woman lived some years longer, it also would have enlarged, and had similar contents.

The tumor above described would seem to be of rare occurrence, as my researches have not enabled me to discover one of

B. *The Anterior Portion of the Uterus.* 1. *The Os Uteri.* 2. *The Fundus.*

3, 3. *The Ovaria and Fallopian Tubes.* 4. *The large Tumor.*

a similar nature. Dr. Baillie mentions a bony mass having been sometimes found in the cavity of the uterus; and gives it as his opinion, that the hard fleshy tubercle of the uterus has, in such cases, been converted into bone.

No uterine disease was suspected during the life of this woman, who had had three children; all her complaints being confined to the chest. A reference to the accompanying sketch will give a better idea of the parts than can easily be conveyed by description.

The following is an account of the chemical composition of this tumor, as contained in a note from Dr. PROUT to Mr. COPLAND HUTCHISON:—"The substance you were kind enough to furnish me with, is more nearly allied to bone than to any thing else that I am acquainted with. When digested in a weak acid for a considerable time, I found that the earthy matter was taken up, and that an animal substance analogous to cartilage was left behind. The earthy matter consisted principally of phosphate of lime. Besides these two principles, there was a portion of fatty matter present. In short, this bony tumor seems to resemble exactly one which was extracted from a wen in the neck, and which is now deposited in the College of Surgeons, together with an account of its composition, &c. as ascertained by myself a few years ago."

### No. II.—*Case of Enlargement of the Heart.*

Thomas Platt, a stout muscular man, thirty-three years of age, by trade a coach-plater, about two years since was attacked with dyspnœa, cough, and pain in the chest, by which he was confined to his room for two or three months. The dyspnœa and pain in the chest gradually subsided, and he followed his usual occupations; but complained very much, at times, of pain in the right hypochondrium, and has constantly been affected with a troublesome cough. About six months ago, the pain in the hypochondrium had left him, and all his complaints at that time were referred to the chest. It was about three months before his death that I first saw him: he then complained (to use his own words,) of great uneasiness in the chest, and breathed with much difficulty, especially in the recumbent position, which produced a sensation of suffocation; the pulsation of the radial artery was upwards of 100, and had a sharp vibratory character; his countenance showed the greatest anxiety, but the expression is not easily described,—it is, I believe, if not peculiar to affections of the heart, always present when this organ is affected; his lower extremities were œdematous; and his urine had lately diminished in quantity. The action of the heart was felt distinctly in the scrobiculus cordis and right hypochondrium. With regard to the treatment, purgatives and diuretics appeared to afford the most relief: cupping on the chest relieved the difficulty of breathing for a short time; but neither cupping nor general bleeding removed, in any degree, the vibratory character of the pulse, although it diminished its frequency and fulness. The anasarca of the extremities gradually increased, and soon became general over the whole body. The difficulty of breathing became most distressing; the man expressed a great dread of going to sleep, lest he should be suffocated, which dread continued till the last. A short time before his death, erysipelatous inflammation attacked the scrotum and upper part of the thighs, which quickly terminated in sphacelation; and death soon released him from his sufferings. I examined his body a short time after death, and the following is a description of the appearances:

In each thoracic cavity, nearly a pint of serous effusion had taken place; the lungs were considerably collapsed, from the pressure of the fluid. In the lower portion of the right side of the chest, a large tumor was observed, resembling a second heart covered with its pericardium: this, on examination, was found to be the right lobe of the liver enormously enlarged, which had pressed up the diaphragm, and was nearly on a level with the heart, and in contact with it. This very satisfactorily accounted for the extensive, though very faint, pulsation which was felt in the scrobiculus cordis and right hypochondrium, as every pulsation of the heart must have been communicated, in some degree, to the enlarged lobe of the liver. The heart weighed *twenty-eight ounces*: its length from the base to the apex was *eight inches*, and its measurement across, in the widest part, *six inches and three quarters*. The aorta, at its commencement just above the semilunar valves, was surrounded by scales of osseous matter, forming a complete bony ring; the aortal valves were much thickened, and their corpora secamoidea enlarged to three times their natural size; the left ventricle was nearly twice the size of the right; but the dilatation was more of the passive than of the active kind, as its parietes were not much thickened. The lungs were of a healthy structure. The right lobe of the liver was nearly three times its usual magnitude, but of a firm healthy structure and colour; the spleen was also considerably enlarged. No other diseased appearances were observed, except a small quantity of serous effusion in the cavity of the abdomen.

The symptoms denoting an affection of the heart were so well marked in this case, as to leave no doubt on the minds of those who saw the patient of there being some disease of this organ. The diagnosis, however, is often extremely obscure; and it becomes worthy of consideration, how far the feelings of the patient can be relied upon in assisting us. I have been informed by a practitioner who has paid much attention to the subject, that persons labouring under disease of the heart very frequently request that they may be opened after death, expressing their conviction that something extraordinary will be found. In the case just related, the man expressed the same wish, and constantly declared to those around him, that he felt persuaded that the real nature of his complaint was not known. Although I do not mention this circumstance of the patient's own view of his disease as one to be trusted, still, as it has occurred often, I think it worth mentioning; as, taken in combination with other symptoms, it may possibly lead us to form a more correct diagnosis.

## Figures and Tables

**Figure f1:**